# An Alternative UV–Vis Spectrophotometric Method for Accurate Quantification of Total Selenium in Se-Enriched Microbial Biomass

**DOI:** 10.1021/acsomega.5c07107

**Published:** 2025-11-10

**Authors:** Caroline Schmitz, Daniel Kuhn, Guilherme Schwingel Henn, Geornelie Promesse Mfoutou Massouangui, Vitória da Silva Marquetto, Gabriela Sbardelotto Zannata, Cláudia Schlabitz, Ani Caroline Weber, Sabrina Grando Cordeiro, Gabriela Altenhofen, Lucélia Hoehne, Daniel Neutzling Lehn, Claucia Fernanda Volken de Souza

**Affiliations:** † Food Biotechnology Laboratory, 186081 University of Vale do Taquari - Univates, Lajeado, RS ZC 95914-014, Brazil; ‡ Graduate Program in Biotechnology, 186081 University of Vale do Taquari - Univates, Lajeado, RS ZC 95914-014, Brazil; § Launer Química Indústria e Comércio Ltda., Estrela, RS ZC 95880-000, Brazil

## Abstract

This study validated a UV–visible spectrophotometric method for quantifying total selenium (Se) in Se-enriched microbial biomass using the selenium–iodine reaction with starch. The protocol employs microwave-assisted acid digestion to ensure complete mineralization of organic Se and was tested in two models: selenized *Lactiplantibacillus plantarum* and spent brewer’s yeast (*Saccharomyces* spp.). Key validation parameters, including linearity, detection and quantification limits, precision, and accuracy, were assessed and compared with inductively coupled plasma–mass spectrometry (ICP–MS) and a certified reference material (SELM-1). Reagent combinations and ultrasonic pretreatment durations (0–30 min) were evaluated; ultrasound caused overestimation due to interference with the starch–iodine complex. Temperature effects (5–30 °C) showed optimal results at 20 °C. Recoveries were in the ranges of 97–111% (bacteria) and 99–101% (yeast), confirming the method’s reliability and robustness. This simple, rapid, and cost-effective protocol enables accurate Se quantification in enriched microbial matrices, making it ideal for quality control in food and biotech industries and for research settings without access to advanced tools like ICP–MS.

## Graphical abstract

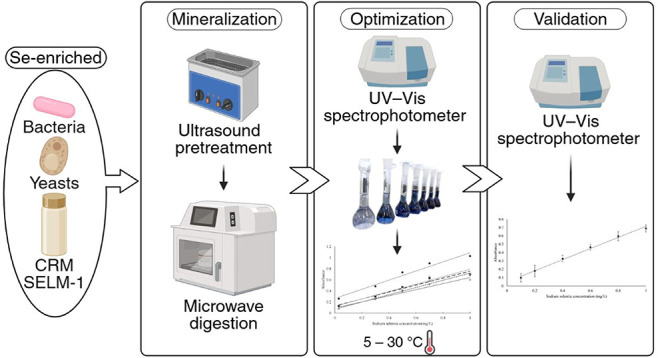

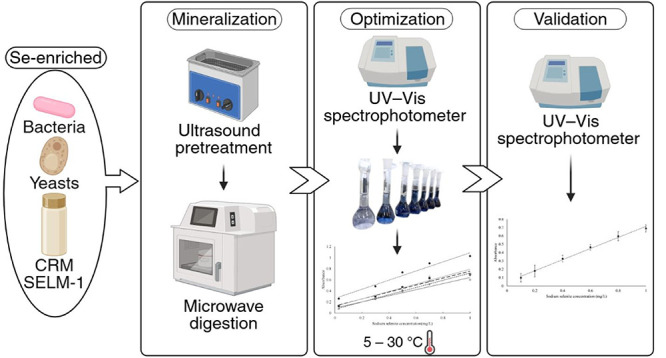

## 1 Introduction

The growing recognition of the physiological importance of selenium (Se) has led to intensified efforts to improve its supplementation given the limited uptake of this element in its inorganic form. A promising approach to enhance selenium bioavailability involves its conversion to organic forms, such as selenomethionine and selenocysteine, which exhibit significantly greater absorption efficacy.^1–3^ Among these, the use of Se-enriched microbial biomass has gained substantial traction in the functional food market, catering to consumer demand for accessible options to improve their dietary intake while providing additional health benefits.^4–10^ This growing demand underscores the need for accessible analytical methods capable of accurately quantifying the selenium content.

The amount of selenium required to achieve its health benefits involves a narrow margin between nutritional efficacy and potential toxicity.^11^ Selenium is an essential micronutrient involved in antioxidant defense, immunological functions, and thyroid hormone regulation.^12,13^ However, its daily intake must be carefully controlled to avoid potential toxicity. For a healthy adult (±70 kg), the World Health Organization^1,2^ recommends a daily intake of approximately 55 μg of selenium, while levels above 400 μg/day may induce toxic effects such as selenosis. Consequently, in addition to the growth of research focused on Se-enriched foods, there is a need to adapt methodologies to ensure adequate quantification during quality control.^14,15^

Selenium biofortification approaches often involve the incorporation of selenized microorganisms, such as Se-enriched lactic acid bacteria and yeast, into functional food products to improve selenium bioavailability. However, ensuring the quality, safety, and regulatory compliance of these products depends on accurate and accessible total selenium quantification methods—whereas current techniques often present limitations in cost, complexity, or equipment availability.^5,6,9^ Despite the robustness and sensitivity of widely used selenium quantification methods such as inductively coupled plasma (ICP) techniques, including ICP–mass spectrometry (ICP–MS) and ICP–optical emission spectrometry (ICP–OES),^16,17^ their high acquisition and operational costs, along with the need for specialized personnel, limit their routine application. These limitations highlight the need for novel, accurate, and cost-effective analytical methods that can ensure quality control without compromising reliability. In this context, UV–visible (UV–vis) spectrophotometric methodologies emerge as compelling analytical options for frequent quality assessments of various food products.^18,19^

Accurate selenium analysis in microorganisms intended for nutritional supplementation relies on the complete mineralization of organic selenium compounds, a critical and often time-consuming step due to the harsh conditions typically required. Modern approaches, such as microwave-assisted digestion, offer significant advantages by enabling the rapid and efficient mineralization of organoselenium species.^20,21^ While the underlying iodine–starch chemistry is well established for bacteria, the novelty of the present work resides in its analytical refinement and explicit validation for microbial matrices. Mörschbächer et al.^22^ previously demonstrated the applicability of a UV–vis method to bacterial biomass; however, that study was limited in matrix diversity, lacked the use of certified reference materials, and did not comprehensively assess pretreatments or operational interferences.

In contrast, the present study aims to improve and validate the iodide–starch chromogenic method for total selenium quantification in Se-enriched microbial biomass. Microwave-assisted digestion was implemented to enhance mineralization, and the influences of ultrasound, temperature, and buffer conditions on triiodide complex stability were systematically evaluated. Validation was performed using selenized *Lactiplantibacillus plantarum*, *Saccharomyces* spp. (spent brewer’s yeast), and the certified reference material (CRM) SELM-1. Key analytical performance parameters, including linearity, efficiency, limit of detection (LOD) and limit of quantification (LOQ), precision, and recovery, were systematically evaluated. The validated method provides a practical and accessible solution for selenium analysis in food, nutraceutical, and biotechnological applications, particularly for laboratories without access to advanced instrumentation such as ICP–MS.

## 2 Results and Discussion

### 2.1 Selenium Mineralization Efficiency in Se-Enriched Lactic Acid Bacterium

Spectrophotometric methods offer advantages over chromatographic techniques in terms of simplicity and cost-effectiveness. However, since this technique targets inorganic selenium, mineralization of (organic) selenium present in samples is a prerequisite. Complete mineralization may be achieved through chemical, physical, or combined processes.^19,23^

Fifteen different digestion protocols were tested (Table 1). Of these combinations, nine were discarded due to high starch oxidation caused by excess reagents (elevated concentrations of nitric acid or hydrogen peroxide in the solutions). This oxidation occurs because, under severe conditions, both reagents act as potent oxidizing agents, degrading the hydroxyl groups in glucose subunits of starch and compromising its helical structure, which hinders the formation of the starch–iodine complex.^24–27^ This conformational alteration in starch prevents chromogenic detection, rendering analytical quantification unfeasible. Therefore, the 1:3 (v/v) combination of ultrapure nitric acid (HNO_3_) and water (H_2_O) was identified as being the most suitable for spectrophotometric analysis.

**Table 1:** Optimization of Digestion Conditions for Se-Enriched Lactic Acid Bacterium: Combinations of Reagents, Pretreatment Step, and Exposure Time to Ultrasound before Microwave Digestion^a^

reagents	preprocess	preprocess time (min)	process	recovery
3.2:3.2:1.6 (HNO_3_:H_2_O_2_:H_2_O)	not applied	not applied	microwave	SO
3:1 (HNO_3_/H_2_O_2_)	not applied	not applied	microwave	SO
3:1 (HNO_3_:H_2_O_2_)	ultrasound	10	microwave	SO
3:1 (HNO_3_:H_2_O_2_)	ultrasound	20	microwave	SO
3:1 (HNO_3_:H_2_O_2_)	ultrasound	30	microwave	SO
3:1 (HNO_3_:H_2_O)	ultrasound	30	microwave	SO
1 (HNO_3_)	not applied	not applied	microwave	SO
2:2 (HNO_3_:H_2_O)	not applied	not applied	microwave	SO
2:2 (HNO_3_:H_2_O)	ultrasound	20	microwave	SO
2:2 (HNO_3_:H_2_O)	ultrasound	30	microwave	SO
4 (H_2_O)	ultrasound	20	microwave	NM
4 (H_2_O)	ultrasound	30	microwave	NM
1:3 (HNO_3_:H_2_O)	ultrasound	not applied	microwave	82–119%
1:3 (HNO_3_:H_2_O)	ultrasound	10	microwave	115–164%
1:3 (HNO_3_:H_2_O)	ultrasound	20	microwave	131–156%

a NM = not mineralized; SO = starch oxidation.

Ultrasound as a mechanical pretreatment alone, followed by microwave-assisted digestion with water, failed to achieve complete mineralization of selenium from its organic to inorganic, quantifiable form (Table 1). Selenium mineralization using only water was ineffective, probably due to its insufficient oxidizing capacity. An acidic environment is crucial to promote oxidation and the complete breakdown of the organic matrix, releasing selenium in a form suitable for analysis.^21,23,24^

Ultrasound pretreatment prior to microwave mineralization was tested for 0, 10, and 20 min in combination with the selected reagents for the acid digestion protocol. Selenium recovery without ultrasound ranged from 82 to 119%, while 10 and 20 min pretreatments yielded recoveries of 115–164% and 131–156%, respectively. These results suggest the formation of byproducts or chemical species during ultrasonic pretreatment that interferes or distorts spectrophotometer quantification, resulting in overestimation of analyte recovery values. Comparatively, microwave digestion alone with no prior treatment yielded values closer to the expected range for accurate quantification at an acceptable range for quality control tests. This reinforces the hypothesis that ultrasound, although effective in prehomogenizing the samples, may introduce artifacts or interferents into the system.^28^

To further validate the methodology for Se-enriched yeast, the same recovery step was applied to the CRM SELM-1 using an identical sample mass (0.6 g). Recovery values for the certified reference ranged from 92 to 115%, at a level deemed acceptable for quantification based on the content provided in the report within European quality control standards. However, further optimizations and refinements are necessary to achieve greater sensitivity of the method.^28^

### 2.2 Performance Optimization of the Starch–Iodine Spectrophotometric Method

The selected methodology is based on the selenium–iodine reaction, with starch serving as a chromogenic agent. Calibration curves were established using sodium selenite standards ranging from 0.01 to 1.0 mg/L. Absorbance was measured at a wavelength of 589 nm using a UV–vis spectrophotometer, with values corrected against a reagent blank (Figure 1).

**Figure 1:**
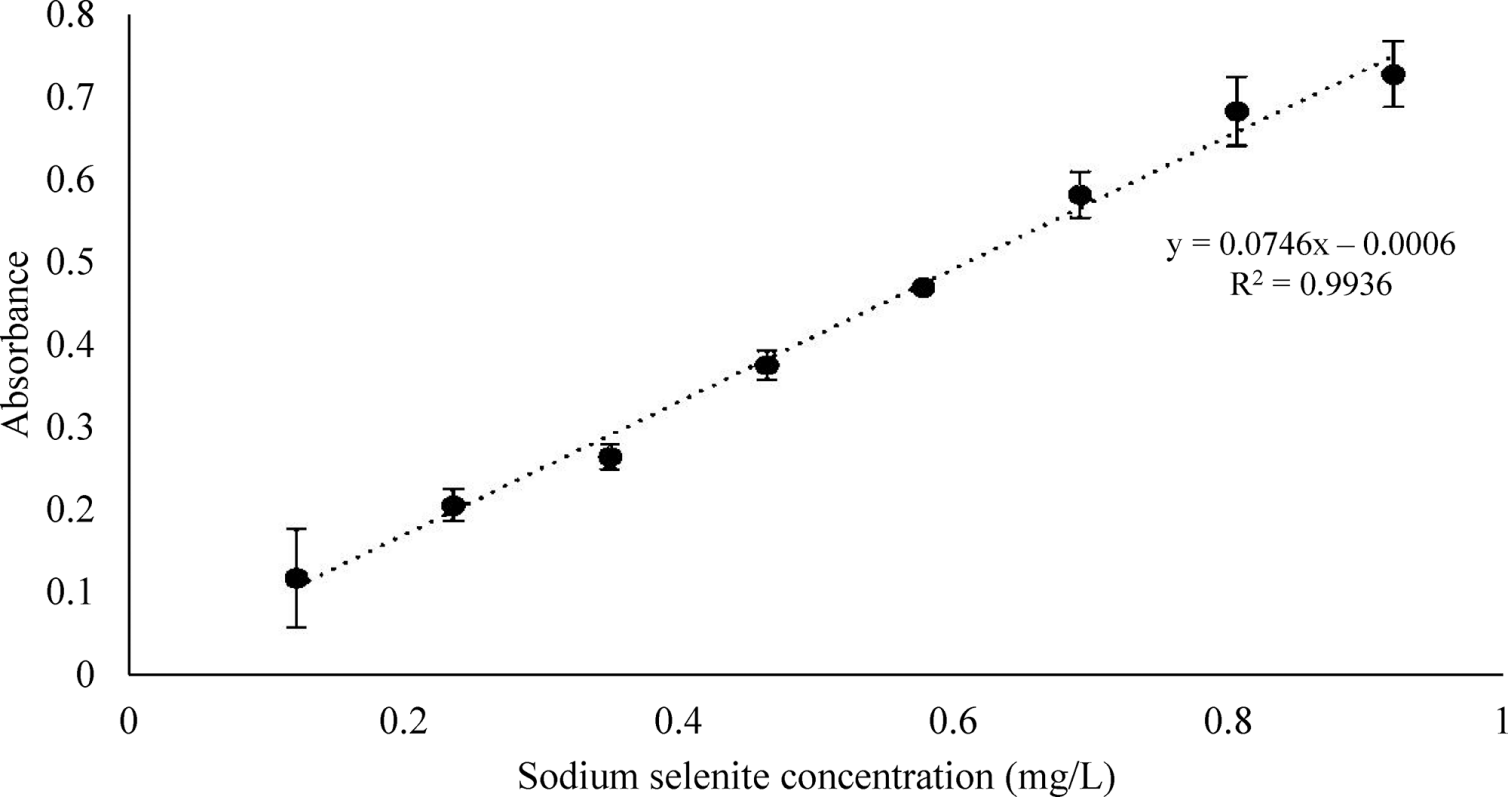
Calibration curve for selenium quantification via the iodine–starch reaction in an acidic medium using a sodium selenite standard (Na_2_SeO_3_).

During initial spectrophotometric analysis, some challenges were identified that warranted optimization: (a) starch solubilization, as inadequate preparation resulted in the formation of granules; (b) reaction kinetics and duration, which proved too rapid for processing more than three samples (*n* = 3); and (c) precipitation at higher selenium concentrations (0.75 and 1 mg/mL) in the sodium selenite standard solutions.

To address starch solubilization and the formation of undissolved granules that can interfere with absorbance, leading to an overestimation of results, different commercial brands were tested. Quemis brand was selected because it provided complete solubilization of starch in 10 mL of ultrapure water (1% w/v) to form a paste at 25 °C, followed by heating to 100 °C for 10 min under constant magnetic stirring (Table 2).

**Table 2:** Effect of Temperature on Analytical Parameters of the Starch–Iodine Model: Relative Standard Deviation (%RSD), LOQ and LOD Values, and Linearity of Calibration Curves

temperature	%RSD	LOQ (mg/L)	LOD (mg/L)	*R* ^2^
5 °C	20–30%	0.067	0.022	0.980
10 °C	1–10%	0.051	0.016	0.984
20 °C	1–11%	0.054	0.017	0.986
25 °C	17–65%	0.064	0.021	0.992
30 °C	25–49%	0.077	0.025	0.981

To optimize analytical throughput, the method was optimized to extend reaction stability for a longer period, with the goal of making it more amenable to industrial application. A 0.3 M acetate buffer solution at pH 4.3 was added immediately after iodine release. This adjustment should slow the reaction kinetics by modulating the strongly acidic pH (initially ∼ 1.3). Instead, operating at pH 4.3 significantly enhances the stability of the iodine/iodide/triiodide system by mitigating the oxidation of iodide by dissolved oxygen, reducing iodine volatilization, and minimizing undesirable side reactions. This controlled pH not only maintains the integrity of the chromogenic complex but also provides a robust and reproducible method suitable for industrial applications.^29^ This pH buffering step enabled the analysis of a larger number of samples simultaneously (*n* = 11), a considerable improvement in practicality for routine use in industry.

Precipitation was observed in the upper calibration range (0.75 and 1 mg/L) at higher temperatures. To assess this effect, reactions were carried out at 5, 10, 20, 25, and 30 °C in a thermal bath (Figure 2). No precipitation occurred at higher selenium concentrations for the reactions carried out at 10–25 °C.

**Figure 2:**
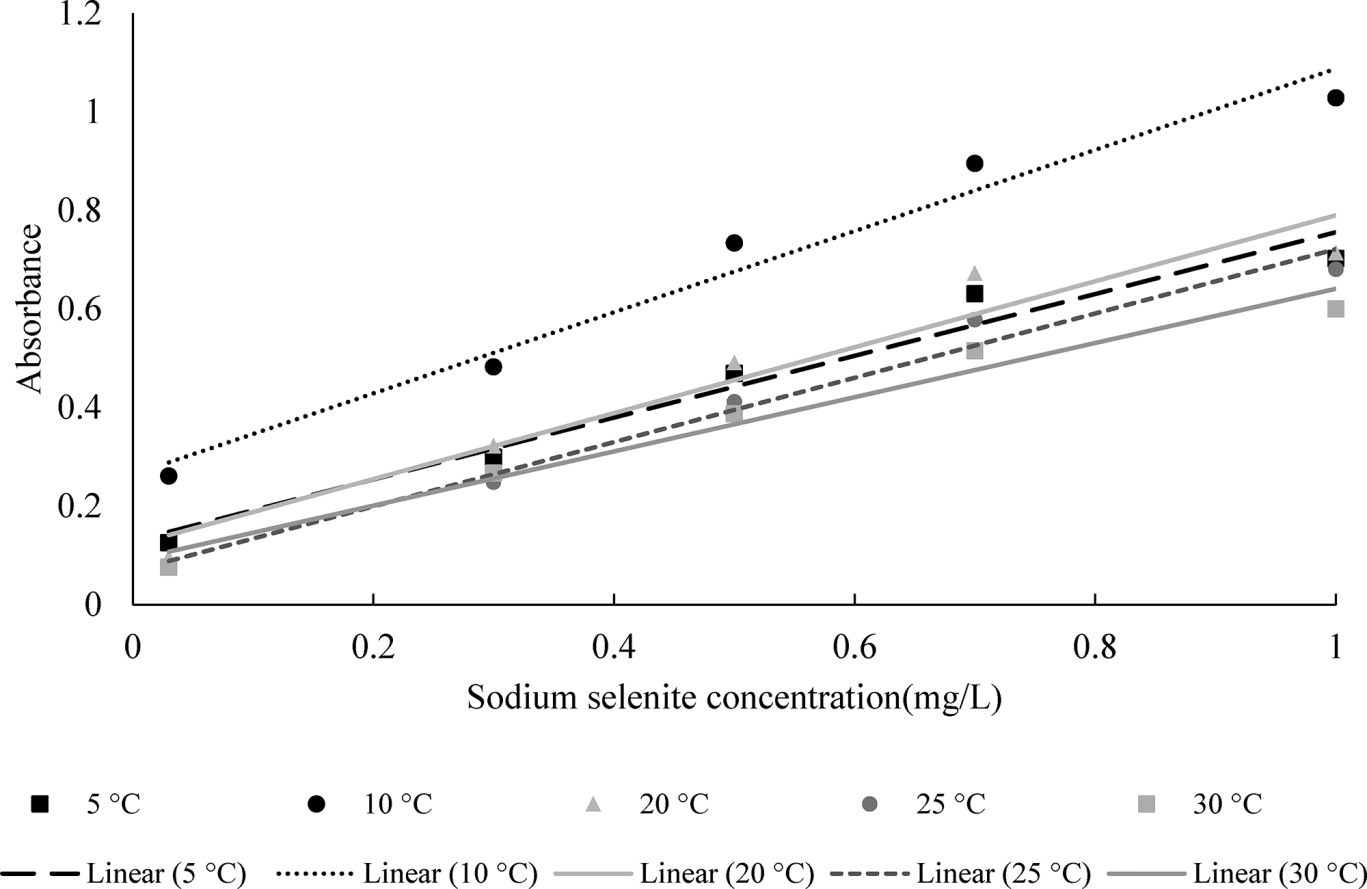
Calibration curve with the linear range of the selenium–iodine reaction, with starch as a chromogenic agent, in an acidic medium for selenium quantification at different temperatures (sodium selenite standard (Na_2_SeO_3_)).

The results demonstrate that temperature significantly influences spectrophotometric quantification when starch is used as the chromogenic agent, corroborating the findings of Hatch and Yang.^30^ The authors reported that the accuracy of the starch–iodine end point is affected by temperature, especially at low analyte concentrations. At 5 °C, high variability was observed, with %RSD values of 20–30%, indicating low method precision. In contrast, intermediate temperatures (such as 10 and 20 °C) showed enhanced stability and precision, with %RSD values ranging from 1 to 11% and improved sensitivity, as reflected by lower LOD and LOQ values. Higher temperatures (25 and 30 °C) led to an increase in variability (%RSD between 17 and 65%) and higher LOD and LOQ, suggesting that high temperatures may compromise the stability of starch–analyte interactions in solutions. These results highlight the importance of strict temperature control to ensure precision and accuracy in spectrophotometric methods employing starch. Considering the economic and technical viability of applying the method, a temperature of 20 °C was considered to be the most appropriate.

In the present study, intermediate temperatures (10 and 20 °C) showed the best results in terms of precision (%RSD between 1 and 11%) and sensitivity, reflected in the lowest LOD (0.016–0.017 mg/mL) and LOQ (0.054–0.067 mg/mL) values. These findings align with those of Hatch and Yang,^30^ who demonstrated that maximum sensitivity for iodometric titrations can be achieved by cooling the iodine solution below 20 °C before the incorporation of starch. On the other hand, extreme temperatures (5 and 30 °C) exhibited higher variability and lower precision and accuracy (i.e., higher %RSD and increased LOD/LOQ). This can be explained by the disruption of interactions between amylose and the triiodide ion (*I*_3_^–^), given that the formation of the blue starch–iodine complex depends on factors such as the reaction temperature and stoichiometry. The stability of the amylose–iodine complex relies on the helical conformation of amylose and the arrangement of triiodide ions along the helix axis, which can be affected by temperature.^31^

Furthermore, these observations reinforce that temperature control is a critical parameter for obtaining consistent and reliable results in analytical methods based on the starch–iodine complex. Temperatures above 25 °C were found to be not suitable due to increased variability and compromised linearity. These findings have important practical implications, especially in the determination of low analyte levels, where significant errors may arise at temperatures exceeding 20 °C.^31^ Future studies could explore the interaction of starch with other chromogenic substances under different experimental conditions to broaden our understanding of the effect of temperature in similar systems.

### 2.3 Protocol Validation

Complex matrix interference is a common challenge in spectrophotometric quantification, particularly when analyzing complex biological samples such as selenized yeasts. These matrices may contain compounds that absorb light at wavelengths similar to those of the analyte, potentially producing overlapping signals that compromise both precision and accuracy.^32^ A standard strategy to minimize these effects is to construct the calibration curve using a sample matrix devoid of Se (i.e., a matrix-matched blank), thereby accounting for the matrix’s specific characteristics and mirroring real sample conditions.^32^ This approach reduces deviations due to matrix effects, enhances calibration accuracy, and ensures that measured concentrations accurately represent actual conditions. Consequently, method validation was performed with and without the matrix, enabling robust comparative analysis. For the bacterial model of this work, matrix simulation was not deemed necessary, as analytical validation metrics—recovery ranging from 82 to 119%—were already within acceptable limits established by the International Council on Harmonisation.^33^

The differing method efficiency observed between the model lactic acid bacterium and spent brewer’s yeast may be related to structural differences in their cell walls and the way in which selenium bioaccumulates. Bacteria such as *Lactiplantibacillus plantarum* have a less complex cell wall, predominantly composed of peptidoglycan and teichoic acids, which may lead to a more superficial and less diversified incorporation of selenium.^32–37^ In contrast, yeast cell walls are composed of a complex matrix of glucans, mannans, and chitin, which function as both a barrier and a selenium reservoir. This structural complexity facilitates the incorporation of selenium species in both organic (such as selenomethionine and selenocysteine) and inorganic forms, through physicochemical binding.^38,39^ These structural and bioaccumulation differences directly affect the extraction and detection of selenium.

The validation process followed the guidelines established by the International Council on Harmonisation,^33^ assessing essential metrics such as LOD, LOQ, linearity, precision, and accuracy. Using starch as a chromogenic agent and sodium selenite as the standard, the calibration curve in the absence of matrix interference demonstrated suitable linearity (*R* ^2^ = 0.99), with LOQ and LOD limits of 0.049 and 0.016 mg/L, respectively. Accuracy was evaluated through recovery tests at three spiking levels using CRM SELM-1 (0.02, 0.12, and 0.32 mg/L) (Table 3).

**Table 3:** Total Selenium Recovery from the CRM SELM-1 Using the Chromogenic Starch-Based Method with and without Matrix Simulation (*n* = 3)

curve	theoretical total selenium (mg/L)	totalselenium quantified (mg/L)	recovery
without matrix	0.024	0.026 ± 0.01	96–110%
	0.124	0.157 ± 0.03	101–127%
	0.323	0.404 ± 0.06	157–174%
with matrix	0.200	0.202 ± 0.01	97–110%
	0.400	0.401 ± 0.01	99–107%
	0.800	0.815 ± 0.02	97–110%

The evaluation of recovery in the absence of matrix components enabled the observation of overestimated results at concentrations near the detection and quantification limits (Table 3). These results highlight the importance of evaluating yeast matrix effects to ensure the reliability and robustness of the analytical method under different conditions. When the yeast matrix was incorporated into the calibration curves, the linearity (*R* ^2^ = 0.99), LOQ (0.05 mg/L), and LOD (0.01 mg/L) showed consistent responses (Figure 3).

**Figure 3:**
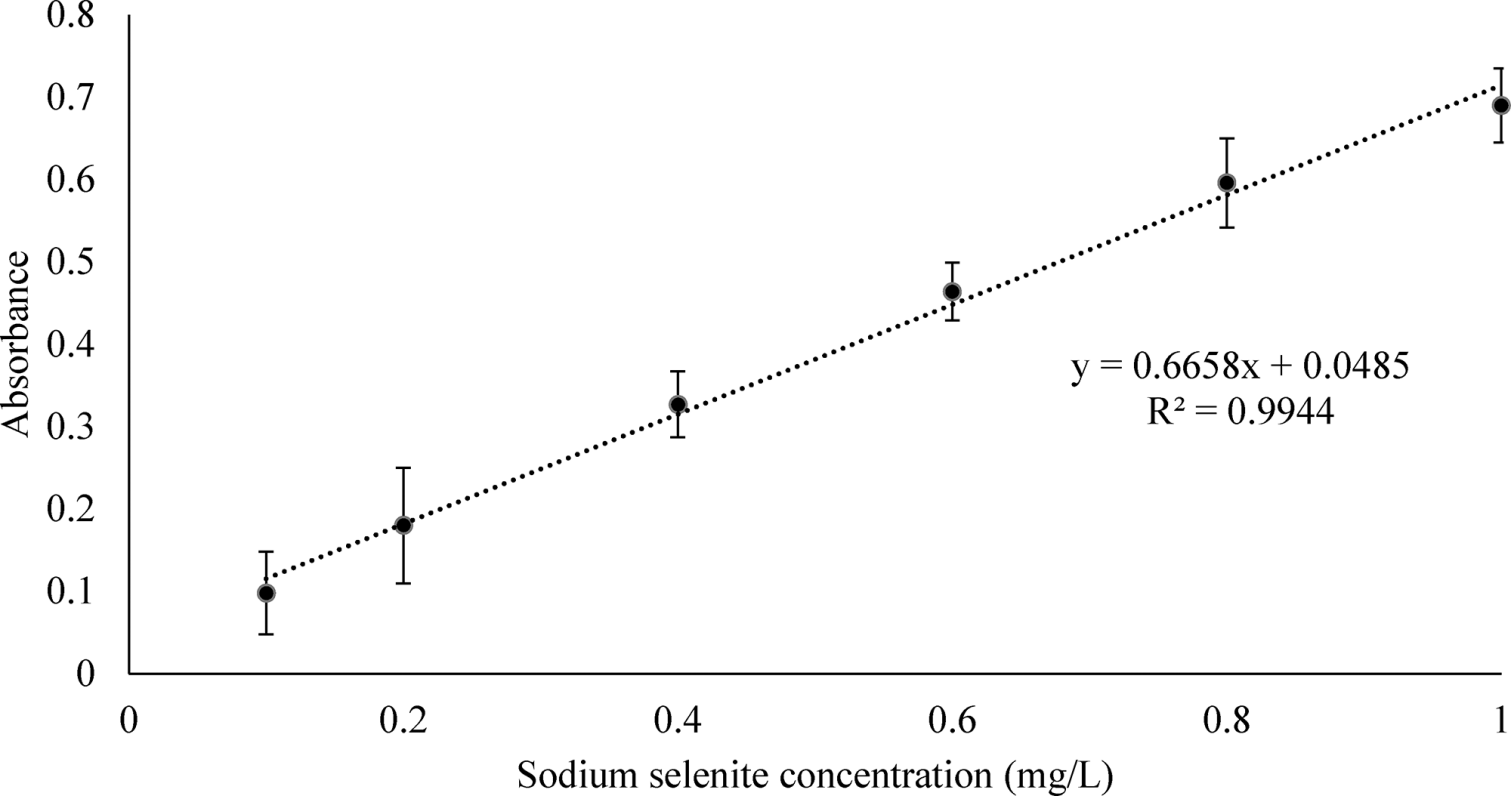
Calibration curve for selenium quantification using starch as a chromogenic agent with matrix correction.

The revised calibration method, which included yeast cell wall matrix simulation, yielded more adequate results in the three analytical recovery points with CRM SELM-1 (Table 3). Accuracy analysis, which considers the recovery at all three tested concentration levels, low (0.20 mg/L), intermediate (0.40 mg/L), and high (0.80 mg/L), exhibited values that met the quality parameters established by the International Council on Harmonisation.^33^

After method validation, total selenium analysis was conducted on both selenized lactic acid bacterium and spent brewer’s yeast. Theoretical selenium content values were obtained through quantification via ICP–MS, which served as a reference method. Samples were digested according to the protocol validated in this study and thus quantified by both methods. Comparisons between ICP–MS and spectrophotometric quantification using starch as a chromogenic agent yielded accuracies ranging from 97 to 111% for bacteria and from 99 to 101% for yeast (Table 4).

**Table 4:** Total Selenium Recovery for Se-Enriched Lactic Acid Bacterium and Spent Brewer’s Yeast Models by the Validated Starch-Based UV–Vis Spectrophotometric Method vs ICP–MS (*n* = 3)^a^

matrix selenized	total selenium quantified by ICP–MS (mg/L)	total selenium quantified by spectrophotometer (mg/L)	recovery
lactic acid bacterium	0.288 ± 0.05	0.260 ± 0.10	97–111%
spent brewer’s yeast	0.489 ± 0.01	0.493 ± 0.08	99–101%

a ICP–MS: Inductively coupled plasma–mass spectrometry.

Although advanced instrumental techniques such as ICP–MS and fluorescence spectroscopy offer inherent robustness and exceptional sensitivity, their widespread implementation for routine quality control and small-scale industrial laboratories remains limited by operational costs and accessibility. ICP–MS is regarded as a benchmark method for total Se quantification due to its ultralow detection limits (in the ppt range) and multielement capability.^17^ However, it demands substantial capital investment and maintenance expenses (e.g., continuous supply of high-purity argon gas) compared to spectrophotometric approaches.^40^ Spectrofluorimetric techniques can achieve comparable or even superior sensitivity than ICP–MS,^41^ but they often depend on the synthesis of complex molecular probes and are prone to fluorescence quenching by selenium or matrix interference in crude biological samples.^42^ In contrast, the UV–vis method validated in this work provides a technically robust and economically feasible alternative for total Se quantification in microbial biomass. Its accuracy was demonstrated to be equivalent to that of ICP–MS, and the method was optimized for high analytical performance through methodological refinements, such as the stabilization of the triiodide complex and buffer control. In alignment with the recognized demand for rapid, low-cost Se quantification protocols in food applications,^17^ the proposed approach meets rigorous analytical standards while emphasizing cost-effectiveness over robust analytical equipment.

The spectrophotometric method reported by Mörschbächer et al.^22^ was instrumental in demonstrating the feasibility of total selenium quantification in bacterial biomass. However, the present study systematically refined the protocol to enhance its robustness, reproducibility, and applicability across diverse complex microbial matrices. The digestion step was optimized, while ultrasound pretreatment was investigated but ultimately excluded due to its tendency to cause significant overestimation of recoveries. Temperature control and the incorporation of an acetate buffer were shown to significantly increase the analytical performance, improving quantification. Furthermore, the validation scope was expanded to include both lactic acid bacteria and spent brewer’s yeast, providing a broader biotechnological context. The inclusion of the CRM SELM-1, together with comparisons to ICP–MS results, confirmed the accuracy and versatility of the improved protocol for routine analyses in laboratories without access to advanced instrumental techniques.

## 3 Conclusions

The UV–vis spectrophotometric method, based on the starch–iodine chromogenic reaction, was improved and successfully validated for total selenium quantification in selenized microbial biomass. Improvements were introduced already in the digestion process, achieved through optimized solvent proportions and the assessment of ultrasound pretreatment. In the quantification step, the reaction mixture temperature was also optimized, and analytical performance was increased by increasing the number of samples processed per batch compared with the method previously described in the literature.

Employing a lactic acid bacterium (*Lactiplantibacillus plantarum*) and spent brewer’s yeast (*Saccharomyces* spp.) as model systems, the method demonstrated excellent performance in terms of linearity, LOD, LOQ, precision, and accuracy. Its simplicity, sensitivity, and low operational cost render it a robust alternative to more complex and expensive techniques, such as ICP–MS, for routine selenium analysis. Its demonstrated reliability and methodological simplicity make it well-suited for efficient selenium determination across diverse stages of production, formulation, and microbial biofortification processes.

## 4 Materials and Methods

### 4.1 Materials

All chemicals, reagents, and culture media were purchased from Merck KGaA (Darmstadt, Germany), Dinâmica (São Paulo, Brazil), Synth (São Paulo, Brazil), and Chemis (São Paulo, Brazil). Method validation was conducted using Se-enriched lactic acid bacteria, yeast, and the CRM SELM-1. A CRM of selenium (isotope ^78^Se, lot BCCG4733, Supelco, Darmstadt, Germany) was used as the calibration standard for ICP–MS.

The lactic acid bacterium used for method optimization was *Lactiplantibacillus plantarum* CH135, supplied by Launer Qumica Indstria e Comércio Ltd. The spent brewer’s yeast (SBY) (*Saccharomyces* spp.) was kindly donated by a brewing industry located in Estrela, Brazil. The Se-enriched yeast CRM SELM-1 was produced by the Institute for National Measurement Standards, National Research Council of Canada (INMS, NRC, Ottawa, Canada). Commercial whey powder, used in encapsulation, was purchased from Silvestre (Três Barras do Paraná, Brazil).

### 4.2 Selenized Microbial Biomass

The lactic acid bacterium *Lactiplantibacillus plantarum* CH135, encapsulated with Arabic gum and maltodextrin, was used as a model for selenized bacteria. Selenization and encapsulation followed the protocol described by Schwingel Henn et al.^43^ Cells were reactivated in Man, Rogosa, and Sharpe (MRS) broth at 37 °C for 24 h until reaching a concentration of approximately 1 × 10^8^ colony-forming units (CFU)/mL. A 10% (v/v) inoculum was then subcultured under the same conditions.

To prepare Se-enriched bacterial cultures, 10% (v/v) aliquots were inoculated in the MRS medium supplemented with 75 mg/L sodium selenite (Na_2_SeO_3_) as the selenium source. Cultures were incubated at 37 °C under constant agitation at 200 rpm (Elo Scientific, Presidente Prudente, Brazil) for 24 h. Growth kinetics were monitored by measuring the optical density at 600 nm using a UV–vis spectrophotometer (UV-2600, Shimadzu, Kyoto, Japan). Upon reaching the stationary phase, microbial cells were separated from the supernatant by centrifugation (CR21GIII, Hitachi, Chiyoda, Japan) at 2540 × g for 20 min at 4 °C. Pellets were resuspended and washed three times with sterile phosphate-buffered saline (0.1 M; pH 7.0).

Encapsulation employed a 1:1 (w/w) mixture of Arabic gum and maltodextrin, with encapsulation solutions formulated at a biomass:encapsulant ratio of 1:20 (w/w). Encapsulation was performed using a spray dryer (MSD 1.0, Labmaq, Ribeirão Preto, Brazil), equipped with a dual-fluid nozzle with a diameter of 1.0 mm, under the following conditions: atomization airflow rate of 40 L/min (5 bar), flow rate solution of 0.5 L/h, drying air flow rate of 1.75 m^3^/min, inlet temperature of 90 °C, and outlet temperature of 75 °C. The Se-enriched encapsulated biomass was collected from the base of the cyclone and stored in sterile flasks.

For the yeast model, selenized SBY (*Saccharomyces* spp.) was used. Raw yeast was preprocessed by sifting through a 35-mesh sieve (Bertel, Caieiras, Brazil) to remove brewing residues, followed by centrifugation (CR21GIII, Hitachi, Chiyoda, Japan) at 3000 × g for 15 min at 8 °C to separate the cells from the brewing liquid. The sediment portion was washed three times with deionized water and centrifuged again under the same conditions.^44,45^

The selenization protocol was adapted from Lomolino and Curioni.^46^ Yeast concentration was adjusted to approximately 1.0 × 1 x 10^7^ CFU/mL in an Erlenmeyer flask (250 mL) containing 100 mL of a liquid medium composed of 1% (w/v) yeast extract and 2% (w/v) glucose. Cultures were incubated for 9 h before a sodium selenite solution (sterilized via filtration through a 0.22 μm filter) was added to the inoculum at a final concentration of 50 mg/L. Incubation continued for up to 30 h at 30 °C. After cultivation, Se-enriched biomass was collected by centrifugation and subsequently freeze-dried (Alpha 1–2 LDplus, Christ, Osterode am Harz, Germany). Primary drying was carried out at 1.0 mbar for 4 h, followed by secondary drying at 0.16 mbar (for 24 h).

CRM SELM-1, derived from selenized yeast, was employed as the validation method. CRM SELM-1 is certified for total selenium (2,031 ± 70 mg/kg), methionine (5,790 ± 100 mg/kg), and selenomethionine (3,190 ± 260 mg/kg). To evaluate industrial scalability, physical and chemical processes were assessed. These consisted of ultrasound-assisted pretreatment, followed by microwave-assisted mineralization using different solvent systems. Optimization tests were performed using Se-enriched *Lactiplantibacillus plantarum* CH135 as the model organism for lactic acid bacteria. The optimal pretreatment was subsequently applied to the CRM SELM-1 and to a sample of selenized SBY (*Saccharomyces* spp.).

### 4.3 Optimized Pretreatment for Selenium Mineralization in Se-Enriched Lactic Acid Bacterium

An ultrasonic bath (SSBu, SolidSteel, Piracicaba, Brazil) operating at 40 kHz was used as a preliminary preparation step to evaluate its efficiency in assisting mineralization prior to microwave digestion. Ultrasound application times of 10 and 20 min were tested with the aim of improving both the mineralization conditions and sample homogeneity.

For each mineralization assay, 0.6 g of a Se-enriched lactic acid bacterium sample was suspended in 6 mL of acidic solvents and placed in a microwave digestion vessel (Multiwave PRO, Anton Paar, Ostfildern-Scharnhausen, Germany). Fifteen combinations of water, nitric acid, and hydrogen peroxide were evaluated in different proportions, and each solvent mixture was tested under ultrasonic pretreatment durations of 0, 10, and 20 min.

Following pretreatment, samples underwent microwave-assisted mineralization under a heating ramp consisting of the following steps: (i) heating to 80 °C over 5 min, (ii) holding for 5 min, (iii) heating to 120 °C over 5 min, (iv) holding for 5 min, (v) heating to 150 °C over 5 min, and (vi) holding for 10 min. After digestion, samples were cooled to room temperature, transferred to 10 mL balloon flasks, and diluted to volume with ultrapure water for Se quantification. Se content in the samples was determined using the spectrophotometric method under validation in this study (Section 4.5) and ICP–MS, serving as the validated reference method (Section 4.6).

### 4.4 Method Development and Calibration for Spectrophotometric Selenium Quantification

A calibration curve was constructed using aliquots of standard sodium selenite solutions ranging from 0.01 to 1 mg/L selenium (linear range test). The protocol was refined and enhanced based on the methods described by Narayana et al.^47^ and Mörschbächer et al.^22^

Reactions were performed in a temperature-controlled bath at 5, 10, 20, 25, and 30 °C. For each standard, 10 mL volumetric flasks were prepared by adding aliquots of selenium standards at 9 different concentrations, followed by the addition of 1 mL of 2% (w/v) potassium iodide solution and 1 mL of 2 M hydrochloric acid to each sample. The mixture was gently shaken until the emergence of a yellowish hue, indicating the release of iodine. After a 3 min incubation period, the reaction was stabilized by adding 1 mL of 0.3 M acetate buffer (pH 4.3). Afterward, 200 μL of 1% (w/v) starch solution was added to the standards as the chromogenic agent, and the final volume was adjusted with ultrapure water. Samples were read after 5 min at 589 nm by using a UV–vis spectrophotometer (UV-2600, Shimadzu, Kyoto, Japan).

### 4.5 Analytical Validation

Method validation for selenium quantification in Se-enriched microbial biomass was performed in accordance with internationally published reference guidelines.^33^ Validation was carried out using the CRM SELM-1 and by comparing results obtained for Se-enriched lactic acid bacterium (*L. plantarum* CH135) and SBY (*Saccharomyces* spp.) samples with those obtained by ICP–MS (see Section 4.6).

Intermediate precision, repeatability, and reproducibility were assessed by using triplicate measurements of three Se solutions with known concentrations established during the linearity test. Overall precision was further confirmed through recovery tests on Se-enriched lactic acid bacterium and SBY samples, with results expressed as relative standard deviation (%RSD) of analyte recoveries. Method robustness was verified by assessing variations in the parameters. Analyses were replicated using a different UV–vis spectrophotometer (Genesys 10S UV–vis, Thermo Scientific, Waltham, USA) and by using potassium iodide and starch reagents from different suppliers (Synth and Qhemis for potassium iodide; Dinâmica and Qhemis for starch).

The calibration curve was constructed by plotting absorbance against Se concentration across a range of 0.010–1.000 mg/L, using ten authentic standard sodium selenite solutions. The final curve represents the average of these individual curves. Linearity was confirmed in accordance with Beer’s law, yielding a molar absorptivity of 1.80 × 10^4^ L/mol^–1^ cm^–1^ at 589 nm based on scans performed on three separate days. LOD and LOQ values were determined from the mean calibration curve and the standard deviation of ten blank measurements, calculated using the equations LOD = 3.3 × (σ/S) and LOQ = 10 × (σ/S).

### 4.6 Total Selenium Quantification by ICP–MS

The Se content in samples of Se-enriched microbial biomass was determined by using ICP–MS (Agilent 7850, Santa Clara, USA). The instrument was operated under the following conditions: a radio frequency generator power of 1.55 kW, an argon flow rate (nebulization) of 1.01 L/min, and an argonium flow rate (plasma) of 4.5 L/min. Quantification was performed using a Se calibration curve ranging from 2.5 to 15.0 μg/L, with an LOQ of 2.5 μg/L.^28^

## Data Availability

Data supporting this study is included within the manuscript.

## Abbreviations

LOD: limit of detection
LOQ: limit of quantification
ICP–MS: inductively coupled plasma–mass spectrometry
CRM: certified reference material
ICP–OES: inductively coupled plasma–optical emission spectrometry
UV–vis: ultraviolet–visible
SBY: spent brewer’s yeast
CFU: colony-forming units
